# A rare cause of Hoffa’s fat pad impingement: the crossed-doubled patellar tendon

**DOI:** 10.1259/bjrcr.20220049

**Published:** 2023-05-25

**Authors:** Charlotte E Munday, Shreena U Patel, Neel Jain, Dina Hikmat, Thomas Armstrong

**Affiliations:** 1 Barnet Hospital Radiology Department, Royal Free London NHS Foundation Trust, London, UK

## Abstract

We present an unusual case of Hoffa’s fat pad impingement syndrome and chondromalacia patellae in the presence of a rare congenital crossed doubled patellar tendon. The crossed-doubled patellar tendon is exceedingly rare. It’s relationship to other conditions involved in anterior knee pain is unclear; however, this case highlights potential pathological associations.

## Clinical presentation

A 34-year-old gentleman presented with an 18-month history of ongoing anteromedial knee pain which they state started following an impact injury whilst playing football. Initial MRI imaging at the time of trauma revealed a Grade 1 intramuscular tear of the proximal vastus lateralis and intermedius and incidentally a crossed-doubled patellar tendon (PT) was noted. The patient was otherwise fit and well with no further significant past medical history or current medication. Clinical examination demonstrated laxity of the medial collateral ligament, however, full range of movement and no joint effusion.

## Differential diagnosis for anterior knee pain

1. Pre-patellar bursitis is inflammation and a collection of fluid within the pre-patellar bursa. This typically arises from chronic irritation due to trauma or repetitive kneeling.^1^ Less commonly, there are associations with rheumatoid arthritis and gout (which can lead to tophi formation within the quadriceps and patella tendons). MRI classically shows a fluid filled sac anterior to the patella demonstrating low T1 and bright T2/short-tau inversion recovery signal which is not shown in this index case.
2. Patella maltracking occurs because of the imbalance of the dynamic relationship between the patella and trochlea during knee motion.^2^ This is often due to an anatomic morphologic abnormality such as trochlear dysplasia, patella alta, lateral patella tilt and/or lateralisation of the tibial tuberosity. Patella maltracking is associated with recurrent patella dislocation (which is usually transient) and more chronic anterior knee pain secondary to patellofemoral cartilage damage, osteochondral defects, and damage to the medial patella stabilisers.^2^ MRI is the imaging modality of choice to assess patella maltracking and the associated, abnormal anatomic features.
3. Patella tendinopathy is a source of anterior knee pain and is commonly characterised by pain at the inferior pole of the patella as a result of repetitive loading on the patella tendon. It is a common condition in young athletes and can be debilitating, often resulting in prolonged absences in sport participation.^3^ The diagnosis of patella tendinopathy remains clinical however imaging such as MRI and ultrasound can be helpful in excluding or including potential alternative diagnoses of anterior knee pain when the clinical picture is not clear.^3^
4. Iliotibial band syndrome is more commonly associated with lateral knee pain following repetitive strenuous physical activity. Fat adjacent to the iliotibial band insertion becomes inflamed. Clinical diagnosis and physical examination is usually sufficient for diagnosis with pain centred over the lateral knee joint or greater trochanter.^4^ MRI typically demonstrates low T1 signal and high T2 signal within the soft tissues lateral to the femoral condyle in keeping with oedema/ fluid.
5. Although less common than ligamentous and meniscal injuries, impingement syndromes of the anterior and posterior suprapatellar fat pads (as well as Hoffa’s (infrapatellar) fat pad as featured in this case) should be considered as causes of anterior knee pain, particularly in active individuals where overuse injury/microtrauma, tendinosis or abnormal patellofemoral alignment may be associated with fat pad impingement syndromes.

## Investigations/imaging findings

**Figure 1.**
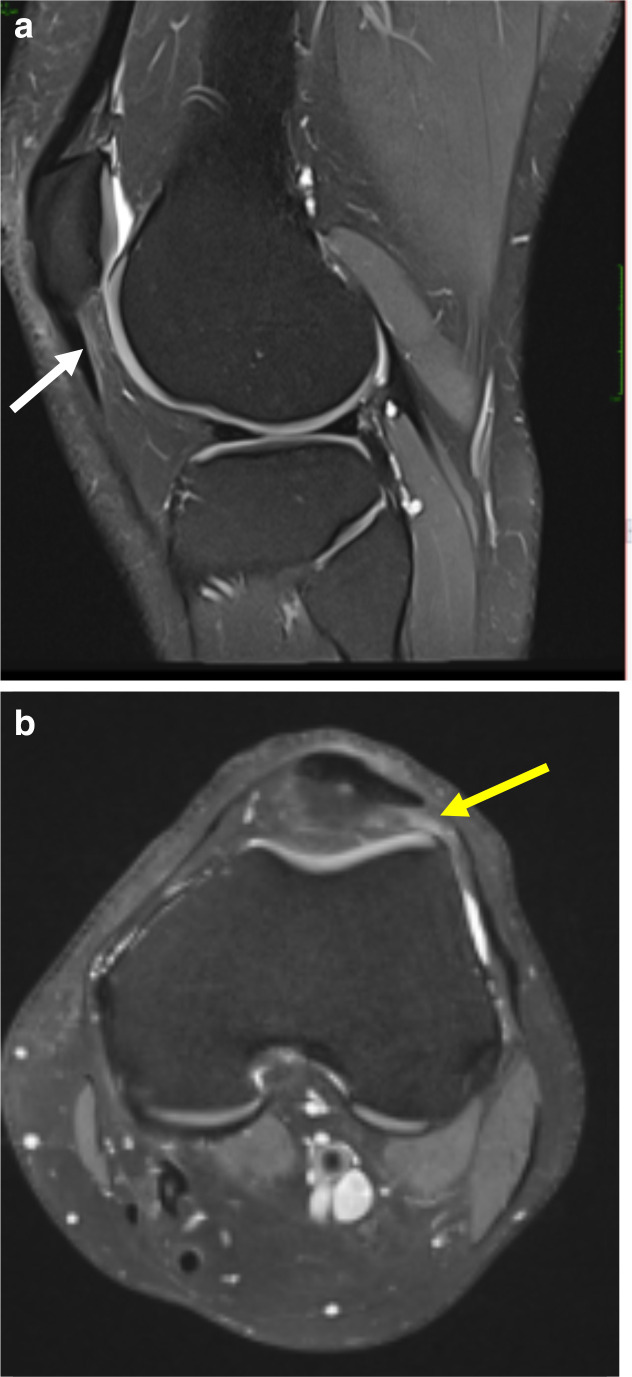
(**a**) Sagittal PD-FS weighted sequence demonstrates a subtle focus of oedema within the superolateral aspect of Hoffa’s fat pad in keeping with impingement (white arrow). (**b**) Axial PD-FS weighted sequence again highlights a focus of oedema at the lateral aspect of Hoffa’s fat pad (yellow arrow). FS, fat-saturated; PD, proton density.

**Figure 2.**
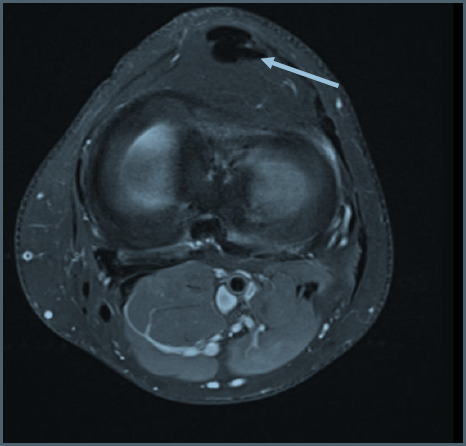
Axial PD-FS image shows the crossed-doubled patella tendon with crossing of the lateral component (from the lateral aspect of the inferior patella) beneath the medial component prior to the insertion on the medial aspect of the tibial tuberosity (arrow). FS, fat-saturated; PD, proton density.

**Figure 3.**
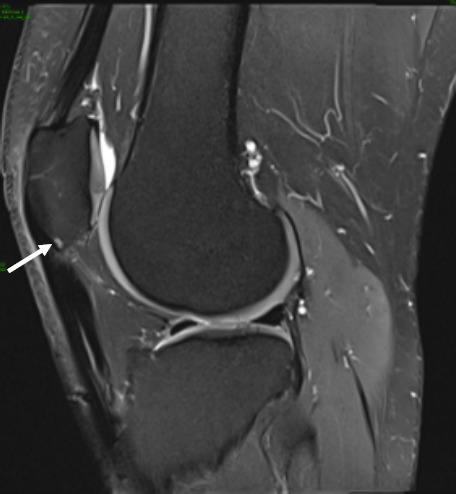
Sagittal PD-FS shows a tiny high signal focus at the inferior pole of the patella which may reflect early tendinosis and corresponding subchondral changes at the insertional fibres of the medial patellar bundle (arrow). FS, fat-saturated; PD, proton density.

**Figure 4.**
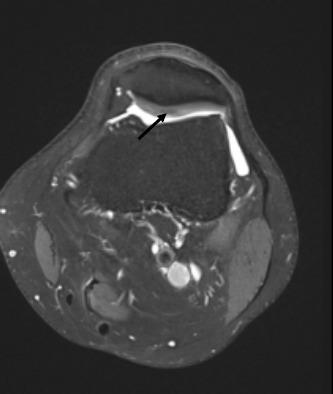
Axial PD-FS image shows partial thickness fissuring of the median ridge in keeping with mild chondromalacia patellae (arrow). FS, fat-saturated; PD, proton density.

- Focal oedema in the superolateral aspect of Hoffa’s fat pad, best appreciated on the sagittal and axial PD-FS (proton density-fat-saturated) MR sequences (Figure 1), with increased signal intensity of the underlying subcutaneous fat. The sagittal T1 sequence confirms subtle oedema with heterogenous reduction of signal in the corresponding location. Radiological findings, along with the patient symptoms of anterior knee pain would support symptomatic Hoffa’s fat pad impingement and early tendinosis.
- The crossed-doubled PT is well appreciated on the axial PD-FS MR sequence (Figure 2), with crossing of the lateral component deep to the medial component named according to their laterality at the patellar origin. The lateral bundle correspondingly inserts onto the medial aspect of the tibial tuberosity and vice versa.
- A small focus of high signal intensity, best depicted on the axial and sagittal PD-FS MR sequences (Figure 3), is at the inferior pole of the patella which suggests very early tendinosis at the patellar origin of the medial bundle.
- Partial thickness fissuring at the median ridge, demonstrated on the axial PD-FS MR sequences (Figure 4), in keeping with mild chondromalacia patellae.
- Normal TT-TG distance.
- Intact ACL, PCL, menisci and collateral ligaments.
- Small joint effusion.

## Treatment

A variety of treatments exist for chondromalacia patellae alone, including non-operative management (reduction of strenuous activities, NSAIDs and exercise to strengthen the quadriceps muscles) and operative (arthroscopic debridement and lavage, articular resurfacing and surgical correction if instability or malalignment). Similar conservative managements of Hoffa’s fat pad impingement syndrome are suggested with specific emphasis on strengthening exercises of the quadricep muscles and proprioceptive exercises of the knee joint to prevent overextension although in rare cases both diagnostic and therapeutic injection can be performed.

## Outcome and follow-up

Following initial assessment in the orthopaedic clinic, the patient was referred to physiotherapy for quadricep strengthening exercises. The patient is currently awaiting a follow-up appointment with the orthopaedic team with the results of his most recent MRI scan.

## Discussion

Anterior knee pain is a common clinical complaint encompassing a broad array of disorders affecting different tissues of the anterior knee compartment.^1^ The anterior knee has been described as having four layers of tissues which are interrelated, meaning pathological conditions can often involve these multiple layers concurrently.^1^

The PT (the second most superficial layer) is a vital part of the knee extensor mechanism (involving the quadriceps tendon, patella, PT and tibial tuberosity) consisting of a band like structure of fibrous tissue connecting the inferior aspect of the patella to the tibial tuberosity.^5,6^ The extensor mechanism of the knee is responsible for extension and patellofemoral stabilisation, with multiple ligamentous structures converging centrally over the patella.^7,8^

The PT is wider superiorly and fibres converge prior to insertion at the superior portion of the tibial tuberosity where it becomes narrowed and thickened.^1^ In the crossover double PT, the PT is composed of two distinct bundles, arising from the anterior surface of the inferior pole of the patella and in this case, demonstrating crossing of the lateral component beneath the medial component prior to tibial insertion.^5^ A double PT is exceedingly rare and noting its existence is important, particularly in cases of pre-operative planning and reconstructive knee surgery (the PT Is favoured frequently as the graft).^7^ As in this case, it is plausible that the crossed-doubled PT may contribute to impingement and resultant anterior knee pain given its different anatomical structure to the single PT, and thus the biomechanics of the extensor mechanism may be altered. This case highlights the potential sequalae of these altered mechanics the crossed-doubled PT may be prone to tendinosis with associated internal impingement; however, this relationship is currently unknown and requires further investigation.^5,9^

The infrapatellar fat pad (Hoffa’s fat pad), is an extrasynovial, intracapsular structure (composing part of the penultimate deep tissue layer of the knee).^1,10^ It is bordered superiorly by the patella, anteriorly by the PT and posteriorly by the femoral condyles.^1^ The peripatellar fat pads prevent friction of the quadriceps and patella tendons with the distal femur and patellar during knee extension and flexion.^11^ Inflammation from chronic overuse and/or repetitive trauma may result in impingement with multiple predisposing factors being suggested. Oedema within the superolateral aspect of Hoffa’s fat pad is typically related to impingement between the PT and lateral femoral condyle in conditions of patellar maltracking.^1^ Markers of patellar instability include increased PT length (with subsequent “high-riding” or “patella alta”), a dysplastic trochlear sulcus (which is often flattened) and increased tibial–tuberosit–trochlear–groove distance. In this case, none of these findings are present that would suggest a common aetiology for the superolateral oedema of Hoffa’s fat pad described in this patient.^12^ Furthermore, Delorme et al have suggested a relationship between proximal patellar tendinosis and patellar maltracking parameters including Hoffa’s fat pad impingement.^13^ This finding is described in this case, depicting a small high signal focus at the inferior pole of the patella, likely reflecting very early tendinosis at the origin of the medial bundle of the crossed-doubled PT.

Chondromalacia patellae is the breakdown and softening of cartilage (an intra-articular structure and part of the deepest of the four tissue layers) on the underside of the patellae. Gursoy et al demonstrated a relationship between superolateral infrapatellar fat pad oedema and patellar maltracking, as well as a significant correlation between patellar maltracking and chondromalacia patellae.^14^ Elevated sulcus angle, lower trochlear depth and patella alta (features that predispose to patellar maltracking) had considerably higher rates of chondromalacia patellae.^14^

In conclusion, there is a significant relationship between Hoffa’s fat pad impingement, chondromalacia patellae and patella maltracking. As the PT plays a key role in the extensor mechanism and partly responsible for patellofemoral stabilisation, it is therefore reasonable to suggest the crossed-doubled PT as seen in this case may be a rare but independent risk factor for maltracking and subsequent impingement and predispose to patella tendinosis.

## Learning points

1. A crossed-doubled patellar tendon is exceedingly rare. It is possible that altered biomechanics and may be a primary cause, or predisposing factor in the development of anterior knee pain.
2. It is important for radiologists to be aware of the crossed-doubled patellar tendon variant so as not to confuse this with previous injury or post-surgical change. It is of particular importance when planning ACL reconstruction using a bone-patella-bone graft.
3. The anterior knee is composed of four layers of tissues which are closely interrelated. Pathological conditions often involve these layers concurrently.
4. Predisposing factors for patella maltracking, in particular an increased higher sulcus angle, shallow trochlear groove and elevated patella height, have a significant relationship with Hoffa’s fat pad and chondromalacia patellae. In the absence of these findings, rarer causes should be considered.
